# Albet H. Fuller, M.D., D.D.S.

**Published:** 1913-01-15

**Authors:** 


ALBET H. FULLER, M.D., D.D.S.—Dr. Fuller died at his home in St. Louis, October 22, 1912, at the age of 71 years. Dr. Fuller was a nephew of Dr. Homer Judd one of the pioneer dentists of St. Louis and the Mississippi Valley. Like his uncle Dr. Fuller was a thorough student of both the science and art of dentistry. He took an active interest in professional matters of his own State and the Nation. He was for many years a teacher in the Missouri Dental College and after the death of Dr. Mudd he became Dean of the school. He was a collector of dental books and a contributor to our journal literature and was highly esteemed in his own city and State as a man of sterling worth and received many honors at the hands of the profession. He was for many years the treasurer of the American Dental Association. He has not been in active practice since 1909. His life was well spent and the dental profession is richer because of his life and labors for it and suffering humanity.

